# DNA methylation biomarkers accurately detect esophageal cancer prior and post neoadjuvant chemoradiation

**DOI:** 10.1002/cam4.5623

**Published:** 2023-01-20

**Authors:** Catarina Macedo‐Silva, Vera Constâncio, Vera Miranda‐Gonçalves, Pedro Leite‐Silva, Sofia Salta, João Lobo, Rita Guimarães, Carina Carvalho‐Maia, Davide Gigliano, Mónica Farinha, Olga Sousa, Rui Henrique, Carmen Jerónimo

**Affiliations:** ^1^ Cancer Biology & Epigenetics Group, Research Center of IPO Porto (CI‐IPOP)/RISE@CI‐IPOP (Health Research Network) Portuguese Oncology Institute of Porto (IPO‐Porto)/Porto Comprehensive Cancer Center Raquel Seruca (Porto.CCC) Porto Portugal; ^2^ Department of Pathology and Molecular Immunology, School of Medicine and Biomedical Sciences University of Porto (ICBAS‐UP) Porto Portugal; ^3^ Cancer Epidemiology Group, Research Center & Department of Epidemiology of IPO Porto (CI‐IPOP)/RISE@CI‐IPOP (Health Research Network) Portuguese Oncology Institute of Porto (IPO‐Porto)/Porto Comprehensive Cancer Center Raquel Seruca (Porto.CCC) Porto Portugal; ^4^ Department of Pathology Portuguese Oncology Institute of Porto Porto Portugal; ^5^ Department of Radiation Oncology Portuguese Oncology Institute of Porto Porto Portugal

**Keywords:** esophageal cancer, liquid biopsies, methylation biomarkers, miRNAs, neoadjuvant chemoradiation

## Abstract

**Background:**

Esophageal cancer (ECa) is associated with high mortality, mostly due to late diagnosis, precluding curativeintent surgery. Hence, neoadjuvant chemoradiation (ChRT) is recommended in most patients regardless of histological subtype. A proportion of these patients, however, achieve complete disease remission and might be spared of radical surgery. The lack of reliable, minimally invasive biomarkers able to detect post‐ChRT disease persistence is, nonetheless, a major drawback. We have previously shown that miRNA promotor methylation enables accurate cancer detection in tissues and liquid biopsies but has been seldom explored in ECa patients.

**Aims:**

Herein, we sought to unveil and validate novel candidate biomarkers able to detect ECa prior and post ChRT.

**Materials and Methods:**

Promoter methylation of *miR129‐2*, *miR124‐3* and *ZNF569* was assessed, using quantitative methylation‐specific PCR (qMSP), in tissue samples from normal esophagus, treatment‐naïve and post‐ChRT ECa, as well as in liquid biopsies from ECa patients.

**Results:**

All genes disclosed significantly different promoter methylation levels between ECa and normal esophagus, accurately detecting post‐ChRT disease, especially for adenocarcinoma. Remarkably, *miR129‐2*_me_/*ZNF569*_me_ methylation panel identified ECa in liquid samples with 53% sensitivity and 87% specificity.

**Discussion:**

*MiR129‐2*_me_, *miR124‐3*_me_ and *ZNF569*_me_ accurately discriminate ECa, either pre‐ or post‐ChRT, from normal tissue, enabling ECa detection. Furthermore, circulalting methylation‐based biomarkers are promising minimally invasive tools to detect post‐ChRT residual ECa.

**Conclusion:**

Overall, our results encourage the use of miRNA methylation biomarkers as accurate ECa detection tools as a novel approach for ChRT response monitoring.

## Graphical abstract

Esophageal cancer methylation biomarkers as suitable tools to discriminate candidates for radical esophagectomy post neoadjuvant chemoradiation.

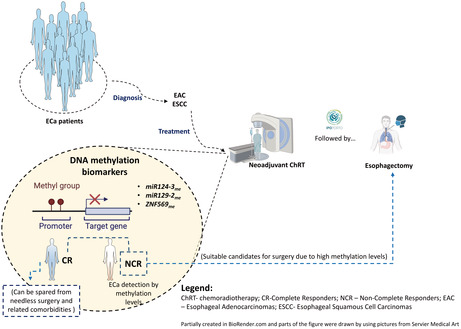

## 1 INTRODUCTION

Esophageal cancer (ECa) ranks as the sixth leading cause of cancer‐related death and the 10th most incident cancer worldwide, considering both genders.^1^ The two most prevalent histological subtypes, esophageal squamous cell carcinoma (ESCC) and esophageal adenocarcinoma (EAC), have distinctive etiologies. Alcoholic and smoking habits are the most common risk factors for ESCC,^1^ whereas obesity, Barrett's esophagus, and gastric reflux disorder contribute for adenocarcinoma.^1^ ESCC accounts for 70% of all diagnosed ECa worldwide, having its highest prevalence in Northern China.^2^ Nonetheless, adenocarcinoma incidence is rapidly growing in high‐income countries.^1^ Although both subtypes are treated following the same guidelines, there is strong evidence that EACs are less responsive to radiotherapy (RT).^2^ Currently, neoadjuvant chemoradiation therapy (ChRT) followed by esophagectomy is standard of care for ECa patients with locally advanced disease (cT3‐T4) of both histological subtypes.^3^ This strategy enables tumor down‐staging before surgery, allowing for improved prognosis.^3^, ^4^ The use of chemoradiation alone with curative intent remains controversial.^5^ According to European Society for Medical Oncology clinical guidelines, definitive ChRT might be considered for locally advanced ESCC patients,^6^ whereas for EAC, either perioperative chemotherapy or neoadjuvant ChRT should be considered standard of care, followed by esophagectomy in patients with locally advanced disease.^6^ Nonetheless, even when complete (R0) tumor response to preoperative ChRT is achieved, patients still undergo surgical procedure.^6^ This is mostly due to the lack of reliable biomarkers which might accurately detect residual disease. In a previous study from our research team, two distinct methylation‐based panels were proposed to accurately discriminate EAC and ESCC tissue samples from normal esophagus.^7^ However, their performance was not evaluated in a post‐ChRT scenario, precisely the situation in which biomarkers would be key to detect residual disease.

In recent years, microRNAs (miRNAs) and their dysregulation through aberrant promoter methylation have emerged as promising cancer detection tools.^8^, ^9^, ^10^, ^11^, ^12^ Indeed, differential miRNAs methylation has been reported in some tumors compared with respective normal samples.^13^, ^14^ Furthermore, miRNAs participate in numerous biological processes, such as cell proliferation, cell death, cell cycle, and associated cellular pathways.^15^ Furthermore, miRNAs DNA methylation levels, in addition to early cancer detection, may forecast patient outcome and response to therapy. Hence, upon careful and comprehensive in silico analysis, we sought to uncover and validate hypermethylated miRNAs promoters as novel ECa biomarkers that might allow for accurate and reliable of disease detection after neoadjuvant ChRT treatment. Remarkably, one of those markers—miR129‐2 methylation (miR129‐2_me_)—has been previously reported in gastric, colorectal, and urothelial carcinomas, as well as in hematological malignancies.^14^, ^16^, ^17^, ^18^ Nonetheless, to the best of our knowledge, methylome‐related studies on this specific miRNA have not yet been reported for ECa, as well as for miR124‐3_me_. Because in our previous study, *ZNF569*_me_ was the best performing biomarker,^7^ it was included in the biomarkers panel tested in the present study.

## 2 METHODS

### 2.1 In silico data analysis: Exploring the most significant CpG sites on miRNAs promoters in ECa

TCGA Human methylation 450 k array in silico data was retrieved from Shiny Methylation Analysis Resource Tool (SMART) App website http://www.bioinfo‐zs.com/smartapp/ to unveil the most relevant specific CpG islands within the promoter region of *miRNAs* genes (Figure S1A). Differential methylation analysis was performed in the TCGA‐ESCA dataset using the following criteria: Hypermethylation, *M*‐Value cut‐off = 2, and Adj. *p*‐value cut‐off = 0.01. CpGs islands and promoter regions were filtered and analyzed as previously described by Lobo and Constâncio et al.^19^ Hence, primers and probes were specifically designed according to the precise localization of the selected CpGs (detailed below). A total of eight miRNAs—miR129‐2, miR124‐3, miR10B, miR663, miR196A1, miR1225, miR34B, miR34C, miR124‐1, miR124‐2, and miR663B—were selected as candidate methylation biomarkers for ECa. Based on the high prevalence of significant hypermethylated (tumor vs. normal) CpG sites at gene promoter region (Table S1), the top two biomarkers (mir‐129‐2 and miR‐124.3) were selected for further testing in our *in house* cohort described below, as well as in an available ESCA‐TCGA dataset. All analyses were performed using RStudio 1.2.5001 for MacOS software.

### 2.2 Patient cohorts and samples selection

A retrospective cohort of 85 ECa patients (46 ESCC and 39 EAC) diagnosed and treated at the Portuguese Oncology Institute of Porto (IPO Porto), between 2007 and 2017 were selected for this study. For the purposes of this study, tumor samples were collected after surgery without previous ChT/RT. All patients were treated in the Digestive Tumors Clinic by the same multidisciplinary team. An additional set of 36 formalin‐fixed paraffin‐embedded (FFPE) tissue samples of ECa following neoadjuvant ChT and/or RT was included. Among these, 20 were found to have complete response (i.e., no evidence of residual disease in the surgical specimen) and 16 with residual tumor. Tumor response to ChT/RT was assessed by a Pathologist with expertise in Digestive Tumors according to the College of American Pathologists templates (Esophagus_4.2.0.0REL_CAPCP), specifically following the proposed Modified Ryan Scheme for Tumor Regression Score, initially proposed for rectal cancer^20^ [(1) single cells or rare small groups of cancer cells–near complete response; (2) residual cancer with evident tumor regression, but more than single cells or rare small groups of cancer cells (partial response); (3) extensive residual cancer with no evident tumor regression (poor or no response)]. Additionally, 57 normal esophagus FFPE samples obtained from total gastrectomy specimens (the esophageal margin segment) without any evidence of ECa were used as controls. Hematoxylin and eosin stained tissue sections were carefully revised and classified by a pathologist with experience on digestive disease, and representative areas were delimitated for further macrodissection.

Additionally, methylation levels of the same genes were assessed in a series of liquid biopsies collected between 2018 and 2021 at IPO Porto, comprising 32 ECa patients (collection prior to any treatment) and 30 asymptomatic controls (Table 1). After collecting peripheral blood into EDTA‐containing tubes, plasma was separated by centrifuging at 2500*g* for 30 min at 4°C and subsequently stored at 80°C in the institutional tumor bank until further use. Relevant clinical and pathological data were retrieved from clinical charts, and an anonymized database was constructed for analysis purposes. This study was approved by the institutional review board (Comissão de Ética para a Saúde) of the IPO Porto, Portugal (CES 202/017). Written informed consent, following the Declaration of Helsinki's ethical principles, was provided by all patients.

**TABLE 1:** Detailed clinicopathological data of ECa patients (FFPE and plasma samples) and healthy donors (plasma)

Clinicopathological data	FFPE tissue samples	Plasma samples	
	ECa treatment‐naive	ECa treatment‐naive	Healthy donors
Patients (no.)	85	32	30
Age median (range)	62 (37–83)	63 (41–89)	62 (50–64)
Sex (no.)			
Man	71	28	14
Woman	14	4	16
Histological subtype (no.)			n.a
Adenocarcinoma (EAC)	39	7	
Squamous cell carcinoma (ESCC)	46	25	
Localization (no.)			n.a
Upper	3	4	
Middle	25	10	
Lower	30	8	
GEJ	27		
T stage			n.a
T1	14	3	
T2	14	2	
T3	55	8	
T4	2	10	
N stage			n.a
N0	37	7	
N1	17	7	
N2	21	2	
N3	10		
N+	–	7	
Stage			n.a
I	11	3	
II	32	5	
III	28	10	
IV	14	14	

Abbreviations: ECa, esophageal cancer; FFPE, formalin‐fixed paraffin embedded; GEJ, gastroesophageal junction; n.a, not applicable; no., number.

A publicly available ESCA‐TCGA dataset retrieved from cBioPortal comprising an in silico series of 15 normal esophagus, 88 EAC, and 95 ESCC tissue samples was also used to assess miRNA methylation levels and the biomarker performance.

### 2.3 DNA isolation and bisulfite treatment and preamplification

Total DNA isolation was performed using FFPE RNA/DNA Purification Plus Kit (Norgen Biotek) for tissue samples following the manufacturer's instructions. Circulating cell‐free DNA (ccfDNA) was extracted from 400 μl of plasma using MagDEA Dx SV kit for the MagLEAD 12gC (Precision System Science Co) automated extractor and eluted in a final elution volume of 50 μl, according to manufacturer's instructions.

Then, bisulfite modification was carried out using 1000 ng of purified FFPE‐derived DNA and the total amount of purified ccfDNA, using EZ‐DNA Methylation‐Gold Kit (Zymo Research), following recommended product protocol. Specifically, two sodium‐bisulfite reactions (25 μl of ccfDNA in each one) were performed per ccfDNA sample in which the final end was a combined eluate volume of 20 μl of bisulfite‐converted DNA per ccfDNA sample case.

Due to the low amount of ccfDNA obtained in liquid biopsies, 8 μl of bisulfite‐treated DNA was pre‐amplified using SsoAdvancedTM PreAmp commercial kit using specific methylation primers for *miR129‐2*, *miR124‐3*, *ZNF569*, and *β‐Actin* genes.

### 2.4 Primer/probe design and quantitative methylation‐specific real‐time PCR (qMSP)

Primers and probes were designed as described in Lobo and Constâncio et al.^19^ All primers, probes, fluorochromes, and quenchers used for this study are listed in Table S2.

Multiplex *miR129‐2*, *miR124‐3*, and *β‐Actin* reactions and *ZNF569* singleplex were performed using methylation‐specific quantitative PCR (qMSP). *β‐Actin* was used as a housekeeping gene. The reaction was carried out in 96‐well plates in ABI 7500 Real‐Time PCR detection system (Applied Biosystems). In brief, 1 μl of bisulfite modified DNA, 5 μl of Xpert Fast Probe mastermix (GRISP) with ROX, an optimized volume of primer (F + R)/probe at 10 μM and sterile bi‐distilled water in a total volume of 10 μl were added to each well. Primer/probe annealing temperature was optimized for all genes at 60°C, except for *ZNF569* which was set at 62°C.

Each sample was run in triplicate. No template controls and negative control (Human HCT116 DKO Non‐Methylated DNA, D5014‐1; Zymo) were included in every plate, assuring the absence of contaminations and specificity for the methylated DNA template. Serial dilutions (five, in duplicate) of positive control (Human HCT116 DKO Methylated DNA, D5014‐2; Zymo) were included in each plate, and a standard curve was computed to assure run efficiency and interpolate comparability. Run efficiency was considered valid when the values were between 90% and 100%. For easier tabulation, final plotting data are represented as the ratio between gene/housekeeping mean quantities multiplied by 1000.

Of note, the results previously published by our group^7^ concerning *the ZNF569* gene were re‐analyzed for post‐ChRT FFPE samples. Thus, the methylation values are derived from a qMSP reaction using Xpert Fast SYBER Mastermix Blue (GRISP) in LightCycler 480 II (Roche, Germany).

### 2.5 Statistical analysis

Non‐parametric tests were used to determine the statistical power of the differences found in methylation levels of the candidate genes among groups. Specifically, the Mann–Whitney *U* test with Bonferroni's correction (when appropriate) and Kruskal–Wallis tests were used to directly compare between two groups or among multiple groups, respectively. Spearman non‐parametric correlation test was used to assess the correlation value between age and methylation levels.

To assess biomarker performance of each gene promoter, samples were categorized as methylated or non‐methylated based on cutoff values established using Youden's J index (value combining highest sensitivity and specificity), through receiver operator characteristic (ROC) curve analysis and calculation of areas under the curve (AUC).^21^ ROC curve analyses were used to assess the biomarker performance in discriminating EAC from normal esophagus or in segregating samples by histological subtype. For the in silico biomarker performance analysis, clinical data was retrieved from cBioPortal and merged with the CpG‐aggregated methylation beta‐values for each gene obtained from SMART App website http://www.bioinfo‐zs.com/smartapp/.

Validity estimates (sensitivity, specificity, positive and negative predictive values, and accuracy) were determined to assess biomarker performance for ECa detection. A panel including selected genes was constructed to improve the detection performance. For that, cases were considered positive if at least one of the genes disclosed a positive methylation value, according to the individual cutoff.

*p*‐value lower than 0.05 was considered as statistically significant. Specifically, *p < 0.05; **p < 0.01; ***p < 0.001; ****p < 0.0001; ns, non‐significant. All data were generated using SPSS Version 27.0.1.0, Chicago, IL and the final representative graphs were constructed using GraphPad Prism software, Version 9.0, USA.

## 3 RESULTS

### 3.1 Clinicopathological features of ECa patients

ECa patient cohort from which FFPE tissues samples were tested included both naïve and post neoadjuvant segregated groups, as well as the normal controls, as detailed in our previous work.^7^ Nonetheless, for the current study, three cases of treatment‐naïve ECa were excluded due to the scarcity of biological material. Updated clinical and pathological data for ECa treatment‐naïve group and for the liquid biopsies' patient cohorts are described in Table 1. No significant differences were found between ECa patients' and healthy donors' median age. Moreover, and except for *miR124‐3*_me_ levels which correlated with age (*R* = 0.169; *p* = 0.016), no other significant correlations were disclosed.

### 3.2 In silico analysis–identification of hypermethylated miRNAs promoters in ECa

To further improve the biomarker performance of our previous reported work,^7^ in silico analysis of CpG sites at CpG islands in miRNAs promoter regions was performed to select additional hypermethylated miRNAs in ECa samples. *MiR129‐2* and *miR124‐3* were disclosed as the top candidate genes based on the number of significantly hypermethylated CpG sites identified in each gene's promoter, six and five hypermethylated CpG sites for miR129‐2 and for miR124‐3, respectively (Table S1). Moreover, miR‐129‐2 and miR124‐3 aggregation methylation levels were also significantly higher in ECa samples compared to those of normal esophagus (*p* < 0.0001; Figure S1B), underpinning their putative value as ECa biomarkers. Although five hypermethylated CpG sites were also found at the promoter region of *miR10b* (Table S1), the low prevalence of CpG sites in the promoter region precluded further analysis by qMSP.

Therefore, miR‐129‐2 and miR‐124‐3 were selected for further validation as ECa screening biomarkers and residual disease detection following ChRT preoperative intervention.

### 3.3 MiRNA promoter methylation levels in treatment‐naïve ECa and normal esophagus tissue samples

**FIGURE 1:**
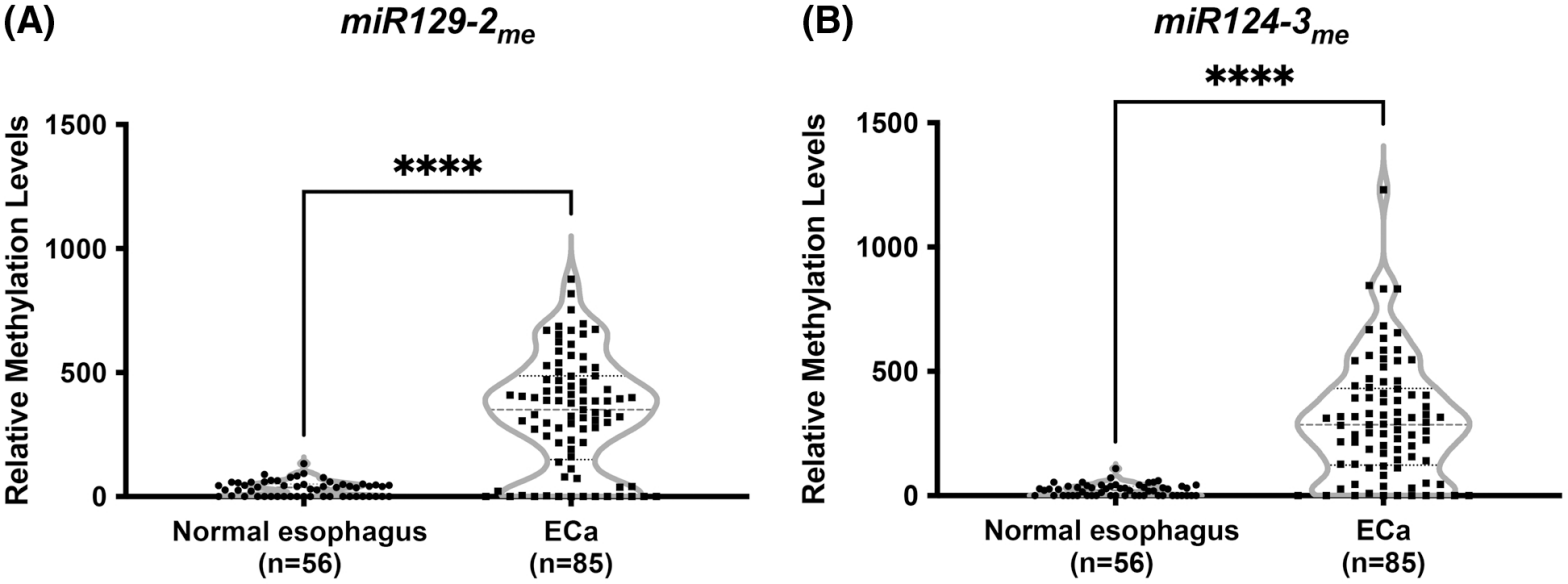
Relative miRNAs promoter methylation levels in naïve ECa tissue samples. (A) *miR129‐2*_me_ and (B) *miR124‐3*_me_ levels were compared between 85 naïve ECa and 56 normal esophagus tissue samples. Both graphs are represented by violin plots with individual dots. Red line represents median values. Median ranks between two groups were compared using the Mann–Whitney test, where **** represents a *p* value lower than 0.0001. ECa, esophageal cancer.

**FIGURE 2:**
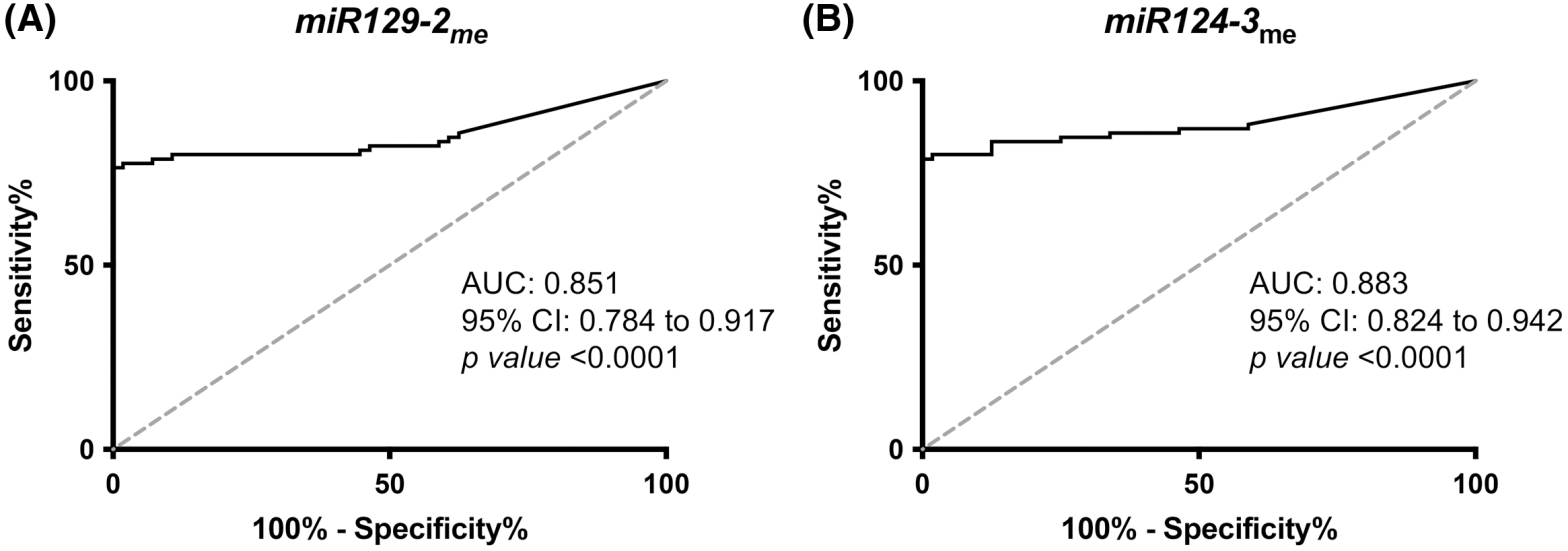
Tissue biomarker performance. Receiver‐operating characteristic (ROC) curve of the (A) *miR129‐2*_me_ and (B) *miR124‐3*_me_ in naïve ECa tissues. ECa, esophageal cancer.

Treatment‐naïve ECa tissue samples displayed significantly higher *miR129‐2*_me_ and *miR124‐3*_me_ levels compared to normal esophagus (*p* < 0.0001 for both; Figure 1). Individually, both *miR129‐2* and *miR124‐3′* methylation levels discriminated cancer from normal esophagus mucosae, with sensitivity values above 76% and specificity of 100% (Table 2; Figure 2). As also performed in our previous study,^7^ we assessed the methylation levels by histological subtype. Although EAC disclosed higher methylation levels compared to ESCC tissues (Figure S2A,B), discrimination was achieved with over 73% sensitivity and 94% specificity (Table 2). Nonetheless, the best biomarker performance was found for both miRNAs in adenocarcinomas (Table 2). Additionally, *miR124‐3*_me_ disclosed the best performance in discriminating EAC from ESCC (AUC = 0.766, Figure S2C) with 74% sensitivity and 73% specificity (Table S3).

**TABLE 2:** Biomarker performance of *miR129‐2*_me_ and *miR124‐3*_me_ for the detection of ECa naïve tissue samples, segregated by histological subtype

Gene	Variables	Sample	Sensitivity (%)	Specificity (%)	PPV (%)	NPV (%)	Accuracy (%)	AUC (95% CI)
*miR129‐2*_me_	Tumor versus normal	FFPE	76.47	100.00	100.00	73.68	85.82	0.851 (0.784 to 0.917)
*miR124‐3*_me_	Tumor versus normal	FFPE	78.82	100.00	100.00	75.68	87.23	0.883 (0.824 to 0.942)
*miR129‐2*_me_	EAC versus normal	FFPE	84.09	100.00	100.00	89.23	93.14	0.897 (0.820 to 0.974)
	ESCC versus normal	FFPE	75.44	96.55	95.56	80.00	86.09	0.836 (0.753 TO 0.920)
*miR124‐3*_me_	EAC versus normal	FFPE	88.64	98.28	97.50	91.94	94.12	0.949 (0.899 to 0.999)
	ESCC versus normal	FFPE	73.21	94.83	93.18	78.57	84.21	0.833 (0.749 to 0.917)

Abbreviations: AUC, area under the curve; CI, confidence interval; ECa, esophageal cancer; EAC, esophageal adenocarcinoma; ESCC, Esophageal squamous cell carcinoma; FFPE, formalin‐fixed paraffin embedded; NPV, negative predictive value; PPV, positive predictive value.

We further evaluated miR‐129‐2 and miR124‐3 methylation levels in the publicly available ESCA‐TCGA dataset. Notably, in agreement with the results depicted in our *in‐house* cohort, the adenocarcinoma subtype had significantly higher methylation levels of both miRNAs, when compared to the squamous carcinomas (Figure S2D,E). Furthermore, both EAC and ESCC groups disclosed significantly higher *miR129‐2* and *miR124‐3′* methylation levels than the normal esophagus group, except for *miR124‐3*_me_ in ESCC (Figure S2D,E). In addition, similarly to our cohort, ESCA‐TCGA dataset biomarker performance in identifying ECa and in discriminating histologic subtypes (EAC vs. normal and ESCC vs. normal) was found, particularly for adenocarcinomas (Table S4). Nonetheless, higher sensitivity in ECa identification (above 90%) was obtained for both miRNAs in this dataset (Table S4).

### 3.4 Biomarker performance in post‐chemoradiation ECa tissue samples

**FIGURE 3:**
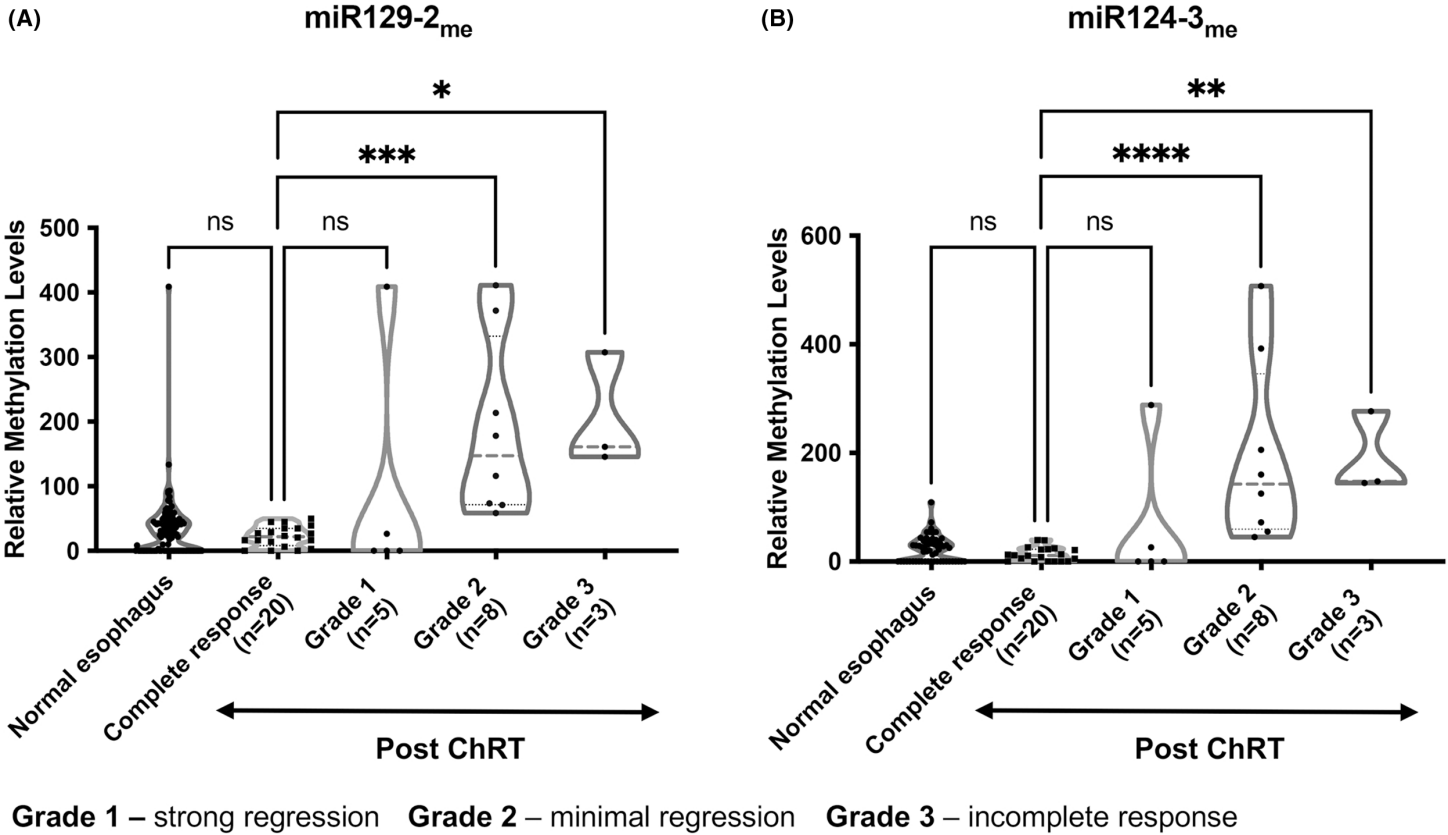
Relative miRNAs promoter methylation levels in post‐ChRT tissue samples. (A) *miR129‐2*_me_ and (B) *miR124‐3*_me_ levels were compared between 16 non‐responders (5 grade 1, 8 grade 2, and 3 grade 3) and 20 complete responders to neoadjuvant ChRT treatment. Furthermore, methylation levels of complete responders with no clinical evidence of disease were compared with normal esophagus (*n* = 56). Both graphs are represented by violin plots with individual dots. Red line represents median values. Median ranks between two groups were compared using the Mann–Whitney test. *p < 0.05; **p < 0.01; ***p < 0.001; ****p < 0.0001; ns, non‐significant. ChRT, chemoradiation.

Because a proportion of patients undergoing ChRT show complete response and might be spared esophagectomy, their identification using accurate biomarkers is of great clinical relevance. Thus, we tested the performance *miR129‐2* and *miR124‐3* methylation levels for detection of residual disease. Interestingly, in tissue samples, increased methylation levels were depicted for both genes in patients with poorer response (grade 2 and 3 scores) compared with those showing complete response (Figure 3). Although a similar trend was found concerning grade 1 response (representing strong regression cases) versus complete responders, it did not reach statistical significance. Conversely, as hypothesized, no significant differences were found between the normal esophageal mucosae and complete responsive samples (Figure 3), which were very low or even null, supporting the hypothesis that these genes are ECa‐specific candidate biomarkers. When stratified by histology, significant differences in methylation levels of both miRNAs between post‐ChRT cases versus complete responders were found for EAC but not for ESCC (Figure S3A,B). This might be due to the predominance of grade 2 or 3 responses in EAC (60% and 30%, respectively), compared to ESCCs, which globally demonstrated better response to ChRT (67% of cases with grade 1 response; Figure S3C). These data agree with previous reports which highlight the better response of squamous‐type carcinomas to ionizing radiation therapy.^2^ Using ROC curve analysis, *miR124‐3*_me_ significantly discriminated incomplete‐responders from complete ChRT responders with 66.67% sensitivity and 100% specificity (Table 3). Moreover, *miR129‐2*_me_ discriminated between grade 1 responders and complete responders, with 20% of sensitivity and 100% of specificity (Table 3).

**TABLE 3:** Biomarker performance of *miR129‐2*_me_, *miR124‐3*_me_, and *ZNF569*_me_ for the detection of ECa post‐ChRT tissue samples

Gene	Variables	Sample	Sensitivity (%)	Specificity (%)	PPV (%)	NPV (%)	Accuracy (%)	AUC (95% CI)	*p* value
*miR129‐2*_me_	IR versus CR	FFPE	66.67	94.44	92.31	73.91	80.56	0.671 (0.469 to 0.874)	0.079
	G1 versus CR	FFPE	20.00	100.00	100.00	81.82	82.61	0.226 (0.000 to 0.493)	0.037
*miR124‐3*_me_	IR versus CR	FFPE	66.67	100.00	100.00	75.00	83.33	0.747 (0.560 to 0.934)	0.011
	G1 versus CR	FFPE	40.00	44.44	16.67	72.73	43.48	0.349 (0.051 to 0.647)	0.250
*ZNF569*_*me*_	IR versus CR	FFPE	64.29	89.47	81.82	77.27	78.79	0.848 (0.705 to 0.991)	0.001
	G1 versus CR	FFPE	100.00	61.11	30.00	100.00	66.67	0.819 (0.618 to 1.000)	0.050

Abbreviations: AUC, area under the curve; CI, confidence interval; CR, Complete responders; ChRT, chemoradiation; ECa, esophageal cancer; FFPE, Formalin‐fixed paraffin embedded; G1, grade 1 responders; IC, incomplete responders; NPV, negative predictive value; PPV, positive predictive value.

In our previous study, *ZNF569* promoter methylation was the best biomarker for detection of treatment‐naïve ECa, and significantly higher methylation levels were disclosed in EAC non‐complete responders post‐ChRT compared to normal esophagus samples.^7^ Hence, the previously generated data for this gene were re‐analyzed, and its performance as post‐ChRT detection biomarker was evaluated. Interestingly, *ZNF569*_me_ discriminated cases with grade 1 response from complete responders with 100% sensitivity, but limited specificity (Table 3).

Concerning histological subtype, *miR‐129‐2*_me_, *miR124‐3*_me,_ and *ZNF569*_me_ significantly discriminated EAC tissues from samples with no evidence of disease post‐ChRT (Table S5). The best biomarker performance was depicted by *miR‐129‐2*_me_ and *miR124‐3*_me_ with 90% sensitivity and 95%–100% specificity (Table S5).

### 3.5 Biomarker performance of *miR129‐2*_me_ and *ZNF569*_me_ in circulating DNA from plasma samples of treatment‐naïve ECa patients

**FIGURE 4:**
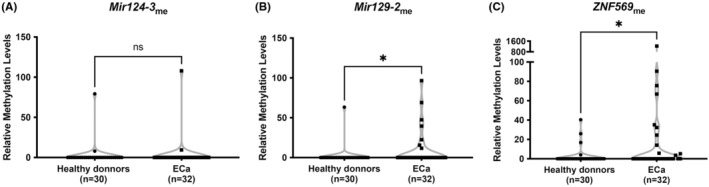
Relative miRNAs promoter methylation levels in plasma samples. (A) *miR129‐2*_me_, (B) *miR124‐3*_me,_ and (C) *ZNF569*_me_ levels were compared between 32 naïve ECa and 30 healthy donors, plasma samples. All graphs are represented by violin plots with individual dots. Red line represents median values. Median ranks between two groups were compared using the Mann–Whitney test. *p < 0.05; ns, non‐significant. ECa, esophageal cancer.

Taking into account the results obtained in tissue samples, the biomarkers were subsequently tested in an independent set of plasma samples, to select the best candidates for accurate and minimally invasive detection of residual tumor post‐ChRT. Thus, performance of *miR124‐3*_me_, *miR129‐2*_me,_ and *ZNF569*_me_ was assessed in plasma samples from ECa patients at diagnosis and healthy blood donors (which served as controls). We found that *miR129‐2* and *ZNF569* methylation levels significantly differed between ECa and controls (Figure 4). Because individual sensitivity of *miR129‐2*_me_ and *ZNF569*_me_ was low (21.88% and 34.38%, respectively), despite >90% specificity (AUC of 0.59 and 0.63, respectively; Table 4) a methylation‐based panel was assembled. Remarkably, this panel discriminated ECa from controls, in plasma samples, with 53.13% sensitivity and 86.67% specificity (Table 4).

**TABLE 4:** Biomarker performance of *miR129‐2*_me_, and *ZNF569*_me_ for the detection of ECa in ccfDNA at diagnosis

Gene	Variables	Sample	Sensitivity (%)	Specificity (%)	PPV (%)	NPV (%)	Accuracy (%)	AUC (95% CI)
*miR129‐2*_me_	Tumors versus AC	Plasma	21.88	96.67	87.50	53.70	58.06	0.592 (0.451 to 0.733)
*ZNF569*_*me*_	Tumors versus AC	Plasma	34.38	90.00	78.57	56.25	61.29	0.630 (0.492 to 0.768)
*miR129‐2*_me_/*ZNF569*_*me*_	Tumors versus AC	Plasma	53.13	86.67	80.95	63.41	69.35	0.592/0.630 (0.451 to 0.733)/(0.492 to 0.768)

Abbreviations: AC, asymptomatic controls; AUC, area under the curve; CI, confidence interval; ECa, esophageal cancer; NPV, negative predictive value; PPV, positive predictive value.

Considering histological subtype, only *ZNF569* methylation disclosed significant differences between EAC and controls, which might be due to the small sample size (*n* = 7 for EAC and *n* = 23 for ESCC, Figure S4A,B). The best biomarker performance was found for EAC (Table S6), and specifically *ZNF569*, with 57.14% sensitivity and 90.00% specificity (AUC = 0.744; Figure S4C; Table S6).

## 4 DISCUSSION

ECa remains a relevant health concern, with estimated 604,000 new cases in 2020 and a cumulative risk of 1.52, ranking 10th in incidence among all cancer types considering both genders, worldwide.^1^ Although incidence has been decreasing, mortality rates remain exceedingly high (544,076 estimated new deaths in 2020, ranking sixth leading cause of cancer‐related death worldwide^1^). Even in Europe, ECa entails a very low 5‐year survival rate (approximately 12%).^22^ The main histological subtypes, EAC and ESCC, emphasize the heterogeneous nature of the disease, reflecting differences in etiology and biogenesis, as well as in tumor location.^22^ EAC incidence seems to be on the rise in developed countries, probably due to increased prevalence of extreme body mass index and/or gastroesophageal reflux.^22^ Nevertheless, ESCC remains the most common subtype (over 70% of cases^2^, ^23^), especially in regions of high incidence, such as Asia.^23^, ^24^ Despite histological diversity, therapeutic strategies are quite similar, with neoadjuvant ChRT followed by surgery recommended for all cases of locally advanced ECa.^3^ Exclusive ChRT is not currently advised and patients proceed to esophagectomy regardless of eventual complete response to neoadjuvant therapy.^6^ This is mostly due to the lack of reliable biomarkers (imaging, molecular) which may accurately indicate whether a complete response was achieved or whether residual disease persists.

The development of such markers would enable discrimination of patients enduring complete response, sparing esophagectomy, with significant benefits. Herein, we sought find and validate hypermethylated miRNAs promoters as novel ECa biomarkers that might allow for accurate and reliable detection of residual disease after neoadjuvant ChRT treatment. Indeed, the detection of post‐ChRT residual disease by non‐invasive means would easily identify patients who need a quick and saving surgical intervention, whereas those without detectable biomarkers would be closely monitored by standard of care procedures along with regular methylation analysis in liquid biopsies.

Remarkably, epigenetic biomarkers such as gene promoter methylation are a promising strategy for early cancer detection, prediction of response to therapy, or disease monitoring after therapy.^19^, ^25^, ^26^, ^27^ Moreover, miRNAs have emerged in the literature as small molecules amenable for the detection in liquid biopsies, being putative diagnostic and/or therapeutic biomarkers for ECa, among other tumor models.^8^, ^28^ Additionally, miRNA promoter hypermethylation may also be used as a cancer biomarker,^14^ and, importantly, have been shown to contribute for cancer radioresistance, limiting its therapeutic efficacy.^29^ To uncover ECa‐specific markers, in silico evaluation looking for methylated miRNAs was first carried out, thus extending our previous work on the identification and validation of DNA methylation‐based biomarkers to early ECa detection and prediction of response to therapy.^7^ Interestingly, from that analysis, *miR129‐2*_me_ and *miR124‐3*_me_ surfaced as candidate biomarkers, significantly discriminating treatment‐naïve ECa tissue samples from normal esophagus, with better biomarker performance than the previously observed for *ZNF569*_me_.^7^ Remarkably, detection performance was maintained in post‐ChRT tissue samples, identifying cases of incomplete response with very high specificity, especially *miR124‐3*_me_ (100%). In previous reports, the tumor suppressive role of a miR124 family member was highlighted,^29^ with significant transcriptional downregulation in ECa tissues versus adjacent normal esophagus samples.^30^ Upregulation, by in vitro transfection, radiosensitized colorectal cancer cells and increased radiation‐induced apoptosis in TE‐1, an ESCC cell line.^30^, ^31^ Importantly, promoter hypermethylation was the regulatory mechanism associated with low expression of hsa‐miR‐124‐3p family members, mediated by the activity of DNA methyltransferase 1 (DNMT1).^32^ Mechanistically, miR129‐2 has also been reported to play tumor suppressive roles by specific targeting *SOX4*.^17^ Moreover, epigenetic inactivation by promoter hypermethylation was shown as a strategic survival mechanism of lymphoid hematological malignancies and a driver for colorectal cancer spread.^17^, ^18^ Nonetheless, most published studies on miRNAs biological function and its implication in ECa radioresistance were only performed in the ESCC subtype.^29^ Thus, our results add significant information to this field, as we included a balanced series of EAC and ESCC.

The miRNAs identified are not exclusive biomarkers of ECa. Notably, *miR124‐3* methylation levels were significantly associated with lymph node metastasis in breast cancer, together with other five miRNAs.^33^ Furthermore, *miR129‐2* promoter hypermethylation, together with another methylated miRNA, miR‐663a, enabled detection of urothelial carcinoma in urine samples.^14^ Moreover, *miR129* promoter methylation was also reported in gastric cancer, hematological malignancies, and metastatic colorectal cancer.^16^, ^17^, ^18^ In breast cancer, it is significantly associated with tumor progression and poor prognosis biomarker.^34^, ^35^ Specifically, cg14416371, a probe within *miR129* CpG island which overlaps the one selected for our study, was previously used to identify pancreatic cancer.^36^ Furthermore, in silico data using two methylation‐based data sets (GSE52068 and GSE62336) identified *miR129‐2* as a candidate hypermethylated gene in nasopharyngeal carcinoma.^37^ Using an in silico approach, *miR129‐2*_me_ in conjunction with other candidate genes, identified oropharyngeal squamous cell carcinoma with 100% specificity.^38^ These data emphasize the relevance of this biomarker for detecting several cancer types. Nonetheless, to the best of our knowledge, this is the first study validating the use of methylated miRNAs as candidate biomarkers for ECa detection.

In our previous study, we compiled a list of methylated genes described in several studies for ECa detection among different sample types, including tissue or liquid biopsies, as well as cytological samples.^7^ After a careful selection, we validated the biomarker performance of *ZNF569*_me_, *GPX3*_me,_ and *COL14A1*_me_ for ECa detection.^7^ Interestingly, differential methylation patterns between EAC and ESCC tissue samples became apparent.^7^ Specifically, two different gene panels significantly discriminated EAC or ESCC tumor samples from normal esophagus, *COL14A1*_me_/*ZNF569*_me_ and *GPX3*_me_/*ZNF569*_me_, respectively.^7^ However, both panels disclosed low specificity (<87.50%) compared to our present results. Nonetheless, the best biomarker performance, enabling accurate ECa detection, was the EAC panel (*COL14A1*_me_/*ZNF569*_me_), with 97.50%sensitivity and 71.43% specificity.^7^ Interestingly, *ZNF569*_me_ was common to both panels, accurately detecting both histological subtypes in combination with other genes. Hence, in our current work, we attempted to verify the performance of the two methylated miRNAs in combination with *ZNF569*_me_, aiming at detection of residual disease in post‐ChRT ECa tissue samples. Globally, the panel disclosed high specificity, with the best performance found for EAC subtype (with or without neoadjuvant treatment), both in tissue and plasma samples. Nonetheless, the highest methylation levels were depicted for *miR124‐3* in EAC. Moreover, *miR124‐3*_me_ significantly distinguished EAC from ESCC naïve tissue samples with superior sensitivity and specificity compared to *miR129‐2*_me_. However, some disparities between plasma and tissue samples are expected considering the different sources. Most plasma samples derived from ESCC patients, which may justify the low detection sensitivity of *miR124‐3*_me_. Moreover, of the two cases detected in plasma by *miR124‐3*_me,_ the highest methylation value was found in one of seven EACs (107.89, *miR124‐3*_me_/*β‐Actin* relative methylation values). Interestingly, most cases that responded poorly to neoadjuvant treatment were EAC (mostly grade 2 and grade 3), which is documented to disclose lower responsiveness to ionizing radiation‐based therapy.^2^, ^39^ Thus, this biomarker may prove especially useful in EAC, for which complete response is less likely to be expected. Although biomarker performance in tissue samples was more robust than in plasma (as expected), it should be emphasized that achieving the goal of sparing complete responders to esophagectomy will require the use of liquid biopsies, because appropriate tissues samples will be more difficult (and hazardous) to obtain and might be less representative. Thus, perfecting residual tumor detection in plasma will constitute the main goal of future studies.

## 5 CONCLUSIONS AND FUTURE PERSPECTIVES

Overall, a robust biomarker (or biomarker panel) enabling accurate discrimination between complete and non‐complete responders to neoadjuvant ChRT in ECa disease is urgently needed, with particular emphasis on EAC, which tends to respond poorly to radiation‐based treatments.^2^ This will allow for sparing radical esophagectomy to complete responders, reducing surgery‐related morbidity and mortality, as well as increasing patients' quality of life. Although our results are based on a relatively small cohort of patients, *miR12*9‐2_me_, *miR124‐3*_me,_ and *ZNF569*_*me*_ provided encouraging results for ECa detection, either prior or post ChRT ECa, which now require further validation and methodological refinement in a larger series of patients, which we are currently recruiting.

## AUTHOR CONTRIBUTIONS

**Catarina Macedo‐Silva:** Conceptualization (equal); formal analysis (equal); writing – original draft (lead). **Vera Constâncio:** Conceptualization (equal); formal analysis (equal); writing – original draft (equal). **Vera Miranda‐Gonçalves:** Conceptualization (equal); formal analysis (equal). **Pedro Leite‐Silva:** Formal analysis (equal); software (equal). **Sofia Salta:** Conceptualization (equal); formal analysis (equal). **João Lobo:** Data curation (equal); resources (equal). **Rita Guimarães:** Methodology (equal). **Carina Carvalho‐Maia:** Methodology (equal). **Davide Gigliano:** Resources (equal); validation (equal); visualization (equal). **Mónica Farinha:** Resources (equal); validation (equal); visualization (equal). **Olga Sousa:** Conceptualization (equal); project administration (equal). **Rui Henrique:** Conceptualization (equal); project administration (equal); supervision (equal); writing – review and editing (equal). **Carmen Jeronimo:** Conceptualization (equal); project administration (equal); supervision (equal); writing – review and editing (equal).

## FUNDING INFORMATION

This study was financially supported by the Prize “Principe da Beira, Ciências Biomédicas 2019” from “Fundação D. Manuel II”, awarded to CM‐S. Additionally, this work was also partially supported by a grant from ESTIMA‐NORTE‐01‐0145‐740 FEDER‐000027. CM‐S, VC, SS, and VMG are recipients of fellowships from, respectively, UniCampania, Naples, Italy (2019‐UNA2CLE‐0170010), “la Caixa” Foundation, Spain (ID 100010434, LCF/BQ/DR20/11790013), Fundação para a Ciência e Tecnologia (FCT), Portugal (SFRH/BD/143717/2019) and the project “P.CCC.: Centro Compreensivo de Cancro do Porto”—NORTE‐01‐0145‐FEDER‐072678, supported by Norte Portugal Regional Operational Programme (NORTE 2020), under the PORTUGAL 2020 Partnership Agreement, through the European Regional Development Fund (ERDF), Portugal.

## CONFLICT OF INTEREST

There are no competing interests to declare in this work.

## ETHICAL APPROVAL

This study was approved by the institutional review board (Comissão de Ética para a Saúde) of the IPO Porto, Portugal (CES 202/017).

## Supporting information


Appendix S1

## Data Availability

Data sharing is not applicable to this article as no new data were created or analyzed in this study.
