# Supplementary material for: DNA methylation biomarkers accurately detect esophageal cancer prior and post neoadjuvant chemoradiation
Source: Cancer Med. 2023 Jan 20;12(7):8777–88. doi: 10.1002/cam4.5623 (PMC10134363; doi:10.1002/cam4.5623)
Supplement: Supplementary file 1 — Appendix S1 [file CAM4-12-8777-s001.pdf]

Macedo-Silva et al.

Supplementary Tables

Supplementary table 1. ***In silico* analysis to explore the most significant CpG sites within promoter region of miRNAs for esophageal cancer.**

Gene	Number of hypermethylated CpGs
miR129-2	6
miR124-3	5
miR10B	5
miR663	4
miR196A1	3
miR1225	2
miR34B	2
miR34C	2
miR124-1	1
miR124-2	1
miR663B	1

Supplementary table 2. Primers/Probe sequences with respective fluorochrome and quencher.

Gene	Sequence	
ß-Actin	**Primers**	F – 5´ TGGTGATGGAGGAGGTTTAGTAAGT 3´
		R – 5´ ACCAATAAAACCTACTCCTCCCTTAA 3´
	**Probe**	5’ Cy5 – ACCACCACCCAACACACAATAACAAACACA – QSY 3’
miR129-2	**Primers**	F- 5’ GGAGTGGTGAGATTGAGTCG 3’
		R- 5’ GACTTCTTCGATTCGCCG 3’
	**Probe**	5’ HEX - CGCGTTGGGGAGATTTAGTTTGTTC 3’
miR124-3	**Primers**	F – 5’ ATAGTCGGTTGAGCGTAGCGTT 3’
		F – 5’ CGTCGAAACCAAAATCGAAAC 3’
	**Probe**	5’ AT 550 – GGCGAAGACGTTTGAGCGTTCG – BHQ2 3’
ZNF569	**Primers**	F – 5’ GAAGTTAGTTTCGTTCGGGTGAGT 3’
		R – 5’ GACACTAAAACCGACGCTATCGAT 3’
	**Probe**	5’ HEX – TGGTGTAACGGATCGAGATTTGTGGCG – BHQ1 3’

Supplementary table 3. Biomarker performance of *miR129-2*$_{me}$ and *miR124-3*$_{me}$ for distinguish EAC from ESCC naïve tissue samples.

Gene	Variables	Sample	Sensitivity (%)	Specificity (%)	PPV (%)	NPV (%)	Accuracy (%)	AUC (95 % CI)	*p value*
miR129-2$_{me}$	EAC vs ESCC	FFPE	66.67	67.39	63.41	70.45	67.06	0.648 (0.529 to 0.768)	0.019
miR124-3$_{me}$	EAC vs ESCC	FFPE	74.36	73.91	70.73	77.27	74.12	0.766 (0.662 to 0.869)	<0.0001

AUC, area under the curve; CI, confidence interval; EAC, esophageal adenocarcinoma; ESCC; Esophageal squamous cell carcinoma; FFPE, Formalin-fixed paraffin embedded; PPV, positive predictive value; NPV, negative predictive value.

Supplementary table 4. Biomarker performance of *miR129-2*$_{me}$ and *miR124-3*$_{me}$ for distinguish ECa naïve tissue samples available at public TCGA database, segregated by histological subtype.

Gene	Variables	Sample origin	Sensitivity (%)	Specificity (%)	PPV (%)	NPV (%)	Accuracy (%)	AUC (95 % CI)	*p value*
miR129-2$_{me}$	Tumor vs normal	TCGA	93.48	86.67	98.85	52.00	92.96	0.858 (0.718 to 0.997)	<0.0001
miR124-3$_{me}$	Tumor vs normal	TCGA	91.85	86.67	98.83	46.43	91.46	0.843 (0.698 to 0.988)	<0.0001
miR129-2$_{me}$	EAC vs normal	TCGA	97.73	86.67	97.73	86.67	96.12	0.881 (0.747 to 1.000)	<0.0001
	ESCC vs normal	TCGA	90.53	86.67	97.73	59.09	90.00	0.838 (0.690 to 0.986)	<0.0001
miR124-3$_{me}$	EAC vs normal	TCGA	96.59	86.67	97.70	81.25	95.15	0.886 (0.752 to 1.000)	<0.0001
	ESCC vs normal	TCGA	87.37	86.67	97.65	52.00	87.27	0.802 (0.642 to 0.962)	<0.0001

AUC, area under the curve; CI, confidence interval; EAC, esophageal adenocarcinoma; ESCC; Esophageal squamous cell carcinoma; FFPE, Formalin-fixed paraffin embedded; PPV, positive predictive value; NPV, negative predictive value, TCGA, the cancer genome atlas.

Supplementary table 5. **Biomarker performance of *miR129-2*$_{me}$, *miR124-3*$_{me}$ and *ZNF569*$_{me}$ for the detection of EAC and ESCC post-ChRT tissue samples.**

Gene	Variables	Sample	Sensitivity (%)	Specificity (%)	PPV (%)	NPV (%)	Accuracy (%)	AUC (95 % CI)	*p value*
***miR129-2*$_{me}$**	EAC (IR) vs CR	FFPE	90.00	95.00	90.00	95.00	93.33	0.817 (0.618 to 1.000)	**0.003**
	ESCC (IR) vs CR	FFPE	42.86	95.00	75.00	82.61	81.48	0.592 (0.260 to 0.923)	0.503
***miR124-3*$_{me}$**	EAC (IR) vs CR	FFPE	90.00	100.00	100.00	95.24	96.67	0.858 (0.676 to 1.000)	**0.001**
	ESCC (IR) vs CR	FFPE	57.14	90.00	66.67	85.71	81.48	0.700 (0.379 to 1.000)	0.144
***ZNF569*$_{me}$**	EAC (IR) vs CR	FFPE	60.00	100.00	100.00	82.61	86.21	0.889 (0.768 to 1.000)	**0.001**
	ESCC (IR) vs CR	FFPE	75.00	68.42	33.33	92.86	69.57	0.658 (0.284 to 1.000)	0.330

AUC, area under the curve; CI, confidence interval; CR, Complete responders; ChRT, chemoradiation; IC, incomplete responders; EAC, esophageal adenocarcinoma; ESCC; Esophageal squamous cell carcinoma; FFPE, Formalin-fixed paraffin embedded; PPV, positive predictive value; NPV, negative predictive value.

Supplementary table 6. Biomarker performance of *miR129-2*$_{me}$ and *miR124-3*$_{me}$ for the detect of EAC and ESCC in ccfDNA at diagnosis.

Gene	Variables	Sample	Sensitivity (%)	Specificity (%)	PPV (%)	NPV (%)	Accuracy (%)	AUC (95 % CI)
miR129-2$_{me}$	EAC vs AC	Plasma	14.29	96.67	50.00	82.86	81.08	0.553 (0.303 to 0.803)
	ESCC vs AC	Plasma	21.74	96.67	83.33	61.70	64.15	0.590 (0.433 to 0.748)
ZNF569$_{me}$	EAC vs AC	Plasma	57.14	90.00	57.14	90.00	83.78	0.744 (0.501 to 0.987)
	ESCC vs AC	Plasma	26.09	87.10	60.00	61.36	61.11	0.569 (0.411 to 0.726)

AC, asymptomatic controls; AUC, area under the curve; CI, confidence interval; EAC, esophageal adenocarcinoma; ESCC; Esophageal squamous cell carcinoma; PPV, positive predictive value; NPV, negative predictive value.

Supplementary figure 1

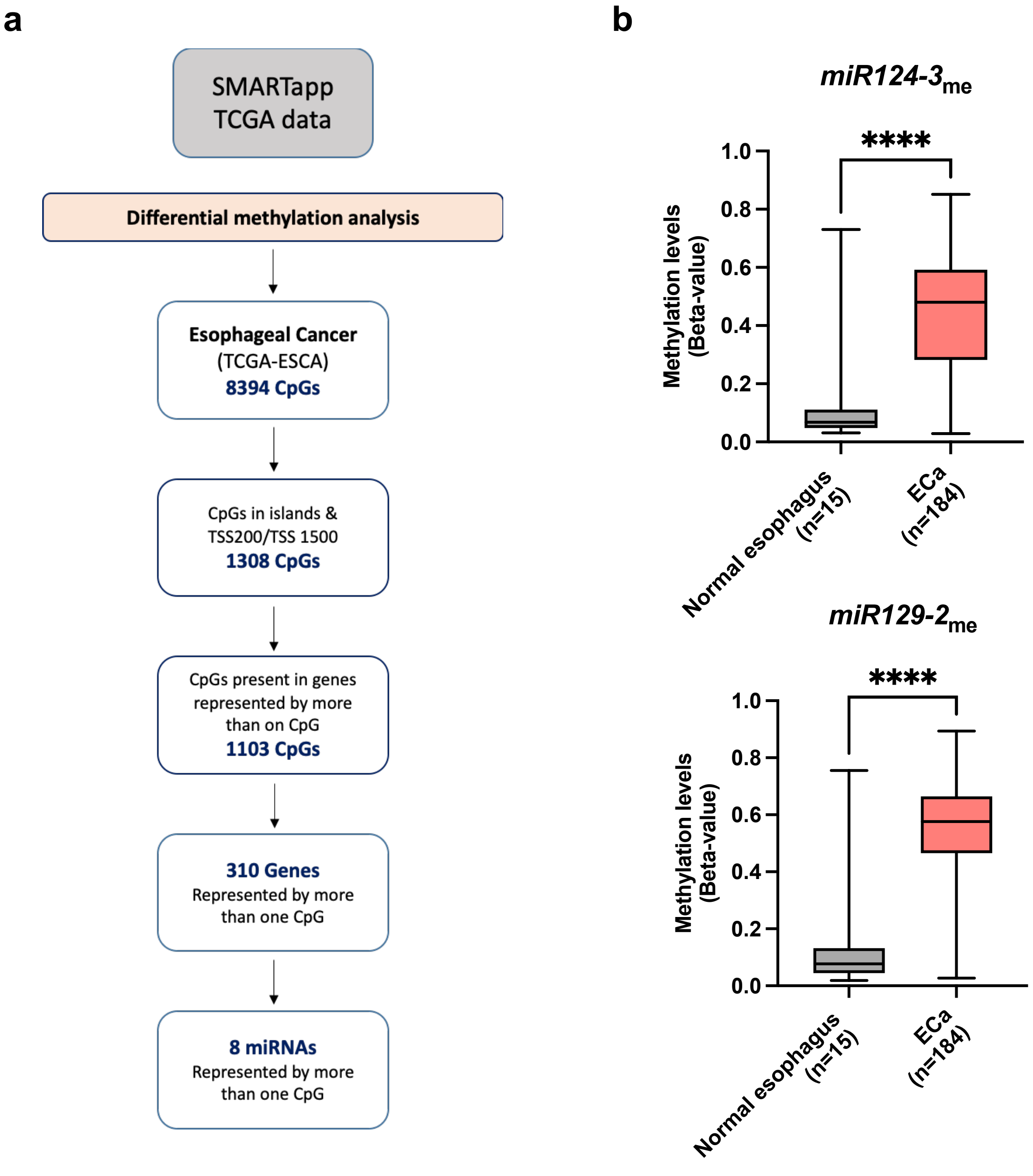

Supplementary figure 2

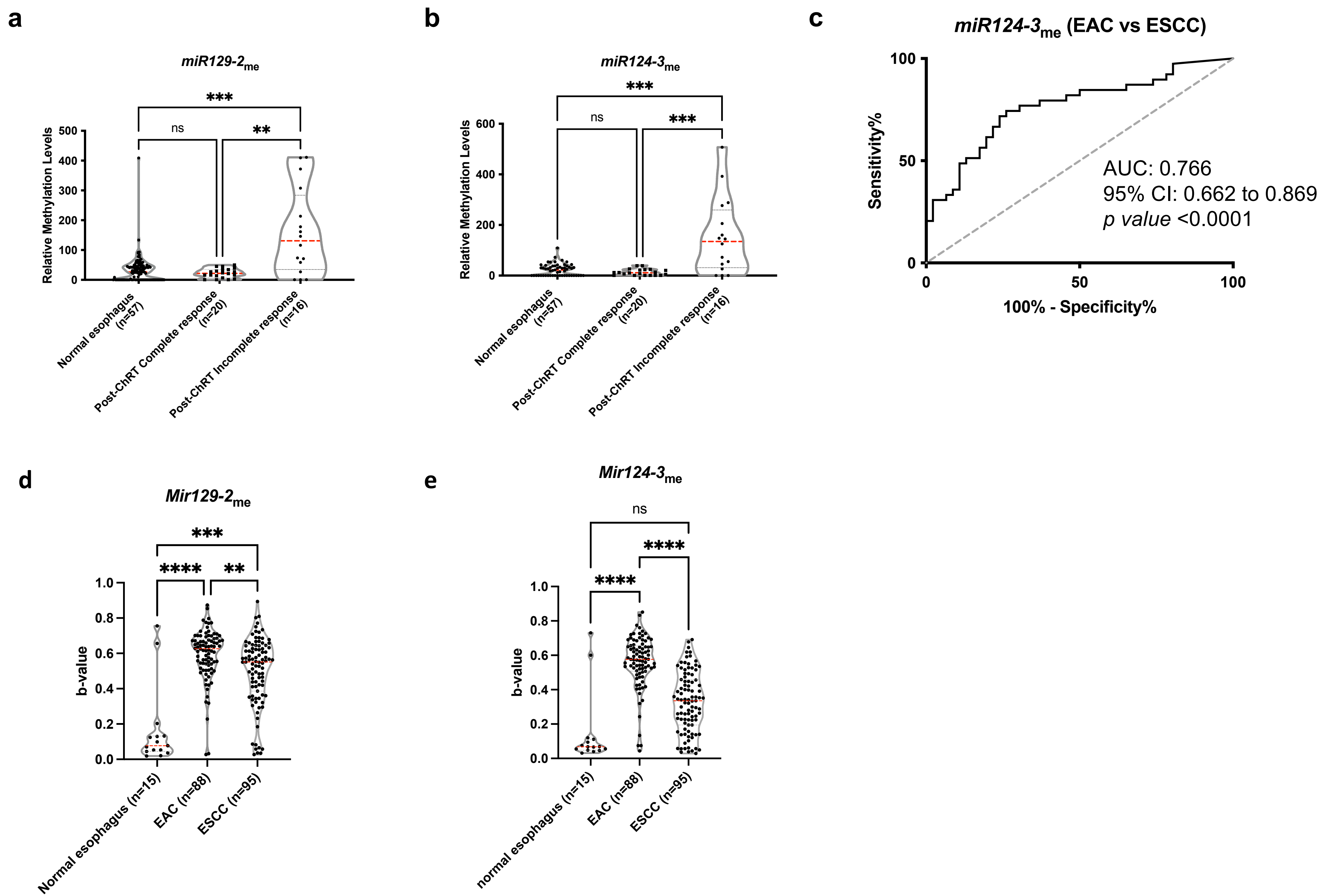

Supplementary figure 3

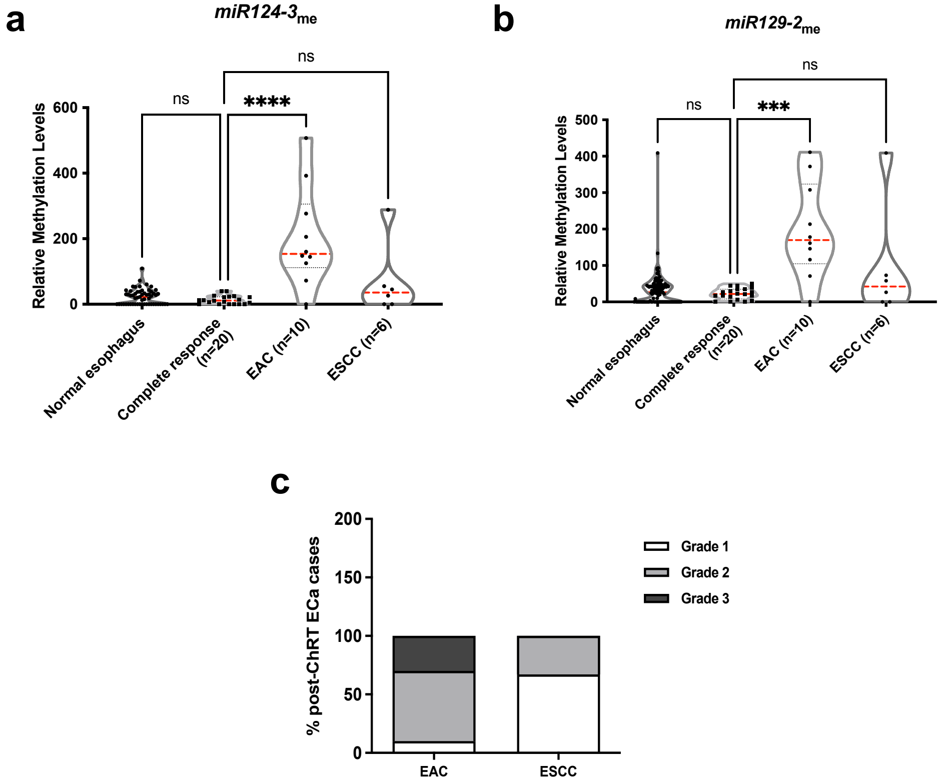

Supplementary figure 4

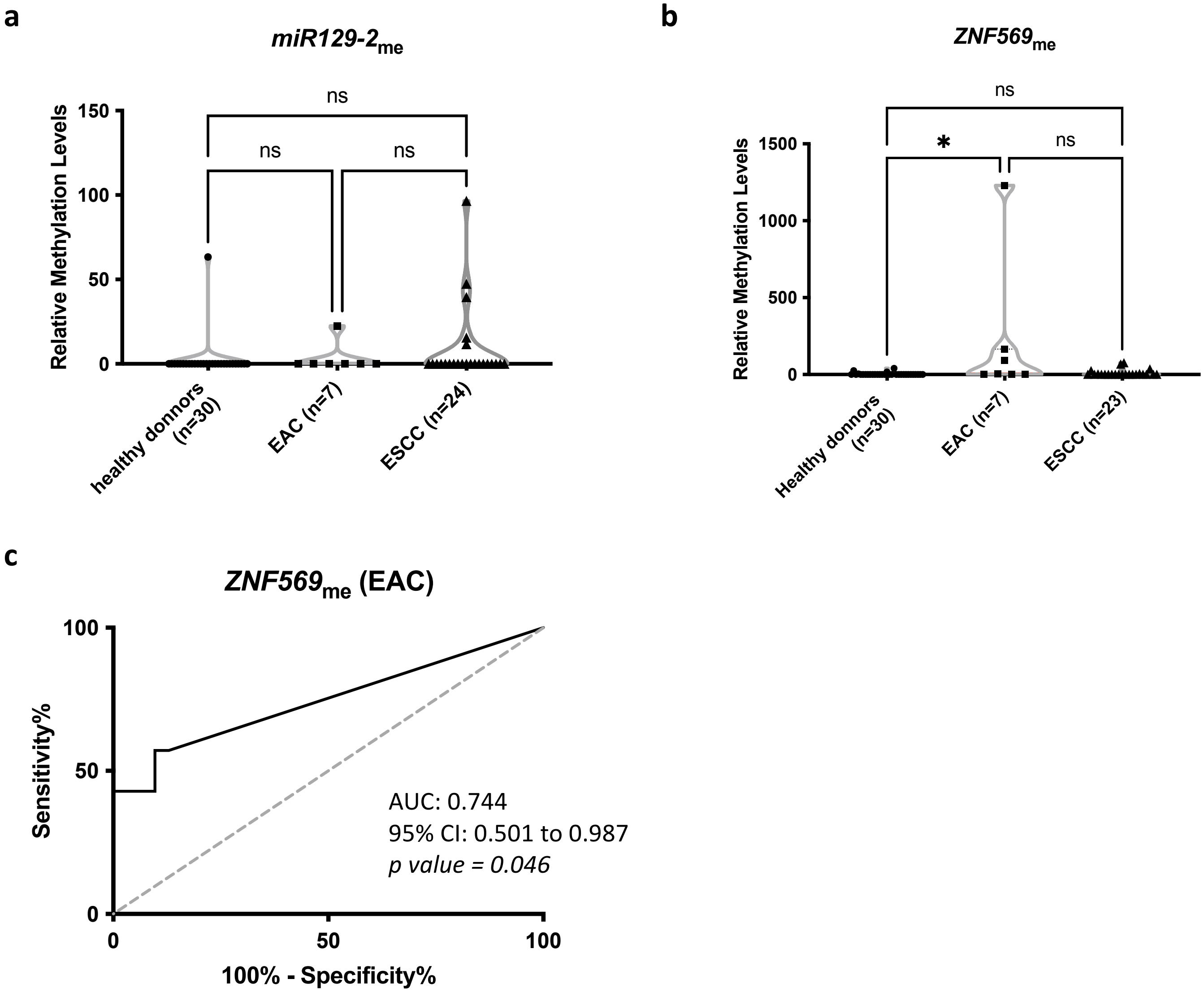

Macedo-Silva et al.

Supplementary figures legends

Supplementary figure 1. In silico analysis for the most relevant methylated CpGs in ECa and normal esophagus. a) Pipeline analysis for differential methylation patterns in ECa. Most relevant specific CpG islands within the promoter region of *miRNAs* genes was assessed using TCGA Human methylation 450k array data retrieved from Shiny Methylation Analysis Resource Tool (SMART) App website http://www.bioinfo-zs.com/smartapp/. **b)** Graphical representation for *in silico miR124-3*$_{me}$ and *miR129-2*$_{me}$ levels in ECa (n=184) and adjacent normal (n=15) tissue samples. Abbreviations: ECa, esophageal cancer; TCGA, The Cancer Genome Atlas.

Supplementary figure 2. Relative miRNAs promoter methylation levels in ECa tissues according with histological subtype. **a)** *miR129-2*$_{me}$ and **b)** *miR124-3*$_{me}$ levels were compared between 46 ESCC or 39 EAC tissues and 56 normal esophagus samples. Red line represents median values. Median ranks between two groups were compared using Mann-Whitney test, where **** represents a *p value* lower than 0.0001. **c)** Biomarker performance of *miR124-3*$_{me}$ represented ROC curve to distinguish EAC from ESCC tissue samples. Abbreviations: ECa, esophageal cancer; EAC, esophageal adenocarcinoma; ESCC, esophageal squamous cell carcinoma; ROC, receiver-operating characteristic. **d)** *miR129-2*$_{me}$ and **e)** *miR124-3*$_{me}$ levels available in the publicly ESCA-TCGA dataset were compared between 95 ESCC or 88 EAC tissues and 15 normal esophagus samples. Clinical data was retrieved from cbioportal and merged with the CpG-aggregated methylation beta-values for each gene obtained from Shiny Methylation Analysis Resource Tool (SMART) App website http://www.bioinfo-zs.com/smartapp/. Red line represents median values. Median ranks between two groups were compared using Mann-Whitney test, where **** represents a *p value* lower than 0.0001.

Supplementary figure 3. Relative miRNAs promoter methylation levels in post-ChRT ECa tissues samples. a) *miR129-2*$_{me}$ and **b)** *miR124-3*$_{me}$ levels were compared between 10 ECa or 6 ESCC non-responders post-ChRT samples and 20 complete responders. Furthermore, methylation levels of complete responders with no clinical evidence of disease were compared with normal esophagus (n=56). Red line represents median values. *** p<0.001; **** p<0.0001; ns, non-significant. **c)** Distribution of grade 1, 2 and 3 post-ChRT non-responders between EAC and ESCC tissue samples. Abbreviations: ECa, esophageal cancer; EAC, esophageal adenocarcinoma; ESCC, esophageal squamous cell carcinoma.

Supplementary figure 4. Relative miRNAs promoter methylation levels in ECa plasma samples. a) *miR129-2*$_{me}$ and **b)** *ZNF569*$_{me}$ levels were compared between 24 ESCC, 7 EAC and 30 healthy donors using plasma samples. *, $p<0.05$; ns, non-significant. **c)** Biomarker performance of *ZNF569*$_{me}$ represented ROC curve to detect EAC plasma samples. Abbreviations: EAC, esophageal adenocarcinoma; ESCC, esophageal squamous cell carcinoma; ROC, receiver-operating characteristic.
